# Anxiety sensitivity and occupational fatigue in meteorological exploration personnel: a serial mediation model of recovery experience and emotion regulation self-efficacy

**DOI:** 10.3389/fpsyg.2026.1803089

**Published:** 2026-03-31

**Authors:** Xiang Ji, Yuchen Yang, Yiya Xu, Yu Zuo, Miaomiao Wang

**Affiliations:** 1 College of Teacher Education, Weifang University, Weifang, China; 2 Shandong Vocational College of Science and Technology, Weifang, China; 3 Guangxi College for Preschool Education, Nanning, China; 4 Xinjiang Key Laboratory of Mental Development and Learning Science, Urumqi, China

**Keywords:** anxiety sensitivity, emotion regulation self-efficacy, meteorological exploration personnel, occupational fatigue, recovery experience

## Abstract

**Background:**

Meteorological exploration personnel work in chronically high-risk and high-demand environments that challenge their psychological well-being and operational safety. Occupational fatigue impairs cognitive function and performance, threatening both mission safety and data quality. Prior research has not fully elucidated the internal psychological mechanisms linking stable traits to fatigue in this population. Grounded in Conservation of Resources (COR) Theory and Self-Efficacy Theory, this study examined the association between anxiety sensitivity and occupational fatigue and tested the serial mediating roles of recovery experience and emotion regulation self-efficacy.

**Methods:**

A questionnaire survey was administered to 311 meteorological exploration personnel. Participants completed the Anxiety Sensitivity Index-3 (ASI-3), Recovery Experience Questionnaire (REQ), Swedish Occupational Fatigue Inventory-25 (SOFI-C), and Regulatory Emotional Self-Efficacy Scale (RESE). Structural equation modeling (SEM) and bootstrap methods were used to test the mediation effects.

**Results:**

The SEM analysis revealed that (1) anxiety sensitivity significantly and positively predicted occupational fatigue; (2) recovery experience mediated this relationship; (3) emotion regulation self-efficacy served as a partial mediator; and (4) a significant serial mediation effect was observed through recovery experience and emotion regulation self-efficacy. Individuals with high anxiety sensitivity reported poorer recovery, which subsequently undermined their confidence in emotion regulation and exacerbated fatigue.

**Conclusion:**

This study provides the first empirical test of a serial mediation model linking anxiety sensitivity to occupational fatigue via recovery experience and emotion regulation self-efficacy among meteorological exploration personnel. Although the relationship was predominantly direct, the significant indirect pathways confirmed the hypothesized mechanistic roles of compromised recovery and eroded self-efficacy in this high-stress context. The findings extend the explanatory scope of COR Theory and Self-Efficacy Theory and provide a nuanced foundation for targeted interventions.

## Introduction

Meteorological exploration personnel are responsible for critical tasks including climate data acquisition, environmental monitoring, and resource surveying. They routinely operate in environments characterized by high physical risk, substantial workload, and considerable uncertainty. Prolonged exposure to harsh natural conditions, demanding operational requirements, and often limited social support subjects this population to persistent psychophysiological stress (Zhang, 2003). The cumulative burden of chronic stress threatens not only individual mental health but may also precipitate occupational fatigue—a multidimensional state characterized by energy depletion, reduced physical capacity, diminished motivation, somatic discomfort, and sleepiness (Ahsberg, 2000). This fatigue impairs cognitive function and operational performance, emerging as a critical factor that compromises mission safety and data integrity (Lee et al., 2016).

Although occupational fatigue is a well-recognized concern in high-risk professions, extant research has largely focused on physiological load or external environmental determinants. A notable gap remains in understanding the psychological mechanisms through which stable individual traits internally initiate and exacerbate fatigue. The present study aimed to delineate the internal process by which specific psychological vulnerabilities, particularly anxiety sensitivity, transform into sustained occupational fatigue within the context of constant occupational stressors.

Anxiety sensitivity (AS), a stable trait defined as the fear of anxiety-related somatic, cognitive, and social-evaluative sensations (Taylor et al., 2007), represents one such vulnerability. According to multidimensional conceptualizations, individuals high in AS tend to catastrophically misinterpret normal physiological arousal as signals of impending loss of control or danger (Reiss and McNally, 1985). In the high-pressure environment of meteorological fieldwork, this trait is pervasively activated: physical discomfort inherent to field operations may be perceived as a serious health threat, while task uncertainty can be amplified into a personal competence crisis. Research indicates that AS is associated with excessive consumption of cognitive and self-regulatory resources, fostering persistent attentional monitoring and rumination that divert mental resources from task execution (Bardeen and Fergus, 2016). Concurrently, AS is related to frequent physiological stress responses. The chronic dysregulation of these responses—particularly within the hypothalamic–pituitary–adrenal axis—may undermine sleep quality and diurnal functioning (Chou et al., 2018). This dual pathway of resource depletion constitutes a core link between AS and fatigue. Therefore, it was hypothesized that anxiety sensitivity would positively predict occupational fatigue among meteorological exploration personnel (Hypothesis 1).

Recovery experience—the process of replenishing psychological and physical resources during non-work time through psychological detachment, relaxation, mastery, or control (Sonnentag and Fritz, 2007)—was posited as a potential mediator. The Effort-Recovery model posits that adequate off-duty recovery is crucial for counteracting resource expenditure from work demands and preventing cumulative fatigue (Meijman and Mulder, 1998). Empirical evidence confirms that high-quality recovery, particularly through detachment and relaxation, mitigates exhaustion and fatigue (Sonnentag et al., 2017). However, this relationship may be disrupted for individuals with high anxiety sensitivity. Drawing on Conservation of Resources (COR) theory (Hobfoll, 1989), high-AS individuals, being in a chronic state of resource loss, may exhibit strong motivation to recover yet are hindered by cognitive patterns such as rumination and hypervigilance, leading to *ineffective recovery*—investing time in rest without achieving psychological replenishment and potentially incurring further resource loss (Sonnentag, 2018). Thus, within this population, recovery experience may function not as a straightforward protective factor but as a complex mediating variable. Accordingly, it was hypothesized that recovery experience mediates the relationship between anxiety sensitivity and occupational fatigue (Hypothesis 2).

To further explicate this mechanism, emotion regulation self-efficacy (ERSE)—defined as perceived capability to manage negative emotions effectively (Bandura et al., 2003)—was introduced as a second mediator. Rooted in social cognitive theory (Bandura, 1997), self-efficacy influences behavior, effort, and perseverance. Individuals with high ERSE are more likely to employ adaptive emotion regulation strategies, conserving cognitive resources (Tamir et al., 2020). Conversely, low ERSE predisposes individuals to maladaptive regulation or avoidance, exacerbating emotional distress and resource depletion. Successful recovery can serve as a mastery experience, enhancing confidence in self-regulatory capabilities and boosting ERSE (Molino et al., 2015). For high-AS personnel, poor recovery outcomes may fail to provide positive mastery feedback and instead undermine confidence in emotion management, leading to reduced ERSE. This erosion of confidence can initiate a vicious cycle in which diminished regulatory self-efficacy makes anxiety more disruptive, further impairs subsequent recovery, and accelerates fatigue accumulation. Therefore, it was hypothesized that emotion regulation self-efficacy mediates the relationship between anxiety sensitivity and occupational fatigue (Hypothesis 3). Given that anxiety sensitivity may also affect occupational fatigue through other unexamined pathways (e.g., sleep disturbance, physiological stress reactivity, or maladaptive coping behaviors), it was anticipated that the direct effect of AS on OF would remain significant even after accounting for RE and ERSE, thus expecting partial rather than full mediation. Furthermore, a serial mediation was posited: recovery experience and emotion regulation self-efficacy operate in a chain mediating sequence between anxiety sensitivity and occupational fatigue (Hypothesis 4), capturing the detrimental process whereby ineffective recovery erodes regulatory confidence.

In summary, no prior study has systematically tested this serial mediating mechanism in the unique context of field-based meteorological exploration. Investigating this integrated model promises to provide a nuanced theoretical perspective on fatigue etiology and pinpoint critical leverage points for targeted interventions, such as recovery skills training or cognitive-behavioral programs designed to enhance self-efficacy.

To provide an overarching framework that integrates these hypothesized pathways, two complementary theoretical perspectives were drawn upon: Conservation of Resources (COR) theory and Bandura’s Social Cognitive Theory. The integration of these frameworks is detailed below.

### Theoretical integration

To provide a coherent framework for the hypothesized model, Conservation of Resources (COR) theory (Hobfoll, 1989) and Bandura’s Social Cognitive Theory (Bandura, 1997) were integrated, offering a clear division of explanatory labor across the proposed pathways. COR theory posits that individuals strive to protect their existing resources and acquire new ones; stress occurs when these resources are threatened or lost, leading to a resource loss spiral (Hobfoll, 2001). In this model, COR theory primarily explains the first two stages of the chain. First, the link between anxiety sensitivity (AS) and recovery experience (RE) is viewed as a resource depletion process. High-AS individuals, characterized by hypervigilance and rumination, experience a chronic drain on their cognitive and emotional resources. This persistent resource consumption directly impairs their capacity to engage in and benefit from recuperative processes such as psychological detachment and relaxation, thereby hindering effective recovery (Hoover et al., 2022). Second, the progression from poor recovery to eroded self-efficacy (RE → ERSE) can also be understood through a COR theory lens. Ineffective recovery represents a failed resource investment; it not only fails to replenish lost resources but can also result in further net resource loss, a phenomenon central to the concept of loss spirals (Hobfoll, 2001). This continued resource depletion undermines the individual’s confidence in their ability to manage future demands, specifically their capacity to regulate emotions.

Bandura’s Self-Efficacy Theory, a cornerstone of social cognitive theory, provides the primary framework for the final pathway from emotion regulation self-efficacy (ERSE) to occupational fatigue (OF). Self-efficacy, defined as the belief in one’s capabilities to organize and execute the courses of action required to produce given attainments (Bandura, 1997), is a key personal resource that influences how individuals think, feel, motivate themselves, and act. Within this framework, ERSE represents a specific, task-relevant belief about managing one’s emotional states. Individuals with strong ERSE are more likely to perceive emotional challenges as manageable, to employ adaptive and resource-efficient coping strategies (such as cognitive reappraisal), and to persist in these efforts, thereby conserving psychological resources (Xanthopoulou et al., 2013). Conversely, individuals with low ERSE doubt their capability to handle emotional distress. This self-doubt can lead them to rely on maladaptive, resource-depleting strategies like catastrophizing, avoidance, or rumination, which directly contribute to the experience of fatigue (Shoji et al., 2016).

The integration of these two theories is therefore complementary and non-redundant. COR theory provides the overarching motivational and resource-based mechanism, explaining why high AS leads to poor recovery and further resource loss. Self-Efficacy theory then elucidates the specific cognitive and behavioral processes—the how—through which this resource-depleted state translates into a detrimental belief system (low ERSE) that ultimately exacerbates fatigue. By combining COR theory’s focus on the dynamics of resource caravans and loss spirals with Self-Efficacy theory’s emphasis on agentic beliefs, the integrated model offers a more comprehensive explanation of the AS-to-fatigue process than either theory could alone, moving from a stable trait, through a resource-depleting process and a compromised belief, to a negative health outcome.

Building on this integrated theoretical framework, the present study aimed to provide the first empirical test of this serial mediating mechanism in the unique context of field-based meteorological exploration. Investigating this integrated model promises to provide a nuanced theoretical perspective on fatigue etiology and pinpoint critical leverage points for targeted interventions.

## Methods

### Participants

Participants were recruited using a cluster sampling approach from field exploration teams affiliated with a provincial Meteorological Bureau and Geological Survey Bureau. The study protocol received ethical approval from the Institutional Review Board of Weifang University. All procedures adhered to the ethical principles of the Declaration of Helsinki.

Inclusion criteria were: (1) current employment as meteorological exploration personnel engaged in field observation, data collection, and equipment maintenance; (2) age ≥ 18 years; (3) provision of informed written consent; and (4) sufficient literacy to complete questionnaires independently.

Exclusion criteria were: (1) > 10% missing data on key variables; (2) patterned or inconsistent responding, judged invalid by two professionals; (3) withdrawal during the survey period.

Of 323 distributed questionnaires, 311 were valid (96.3% response rate). The sample comprised 297 males (95.5%) and 14 females (4.5%). This gender distribution accurately reflects the demographic composition of meteorological field exploration personnel in China, where the physically demanding and remote nature of the work results in a predominantly male workforce (Li et al., 2020; Wang and Zhang, 2018).

Participants’ ages ranged from 20 to 42 years, with a mean age of 29.4 years (*SD* = 4.2). The age distribution was as follows: 20–24 years (*n* = 68, 21.9%), 25–29 years (*n* = 112, 36.0%), 30–34 years (*n* = 79, 25.4%), 35–39 years (*n* = 41, 13.2%), and 40–42 years (*n* = 11, 3.5%).

### Measures

#### Anxiety sensitivity index-3 (ASI-3)

The Chinese version of the ASI-3 (Taylor et al., 2007; Wang et al., 2011) was used to assess anxiety sensitivity. This 18-item scale measures three dimensions: physical concerns, cognitive concerns, and social concerns. Items are rated on a 5-point Likert scale ranging from 1 (very little) to 5 (very much), with higher scores indicating greater anxiety sensitivity. In the present sample, Cronbach’s *α* was 0.98 for the total scale, with subscale *α* coefficients ranging from 0.92 to 0.95. The high internal consistency may be partly attributable to the relatively homogeneous nature of the sample (all participants were meteorological exploration personnel working under similar high-stress conditions). To further assess the potential for item redundancy, the mean inter-item correlation for the total scale was examined; it was 0.62, falling within the recommended range of 0.15–0.50 (Clark and Watson, 1995), suggesting that the items are highly consistent but not merely redundant.

#### Regulatory emotional self-efficacy scale (RESE)

The Chinese version (Yu et al., 2009) was administered. This 11-item scale measures self-efficacy in expressing positive emotions and managing despondency/distress and anger/irritation on a 5-point scale. Higher scores indicate stronger self-efficacy. Cronbach’s *α* was 0.98 for the total scale; subscale *α* ranged from 0.91 to 0.95. The mean inter-item correlation for the total scale was 0.59, which is within the acceptable range (Clark and Watson, 1995), indicating that the high alpha reflects true internal consistency rather than problematic item redundancy.

#### Swedish occupational fatigue inventory-25 (SOFI-C)

Subjective fatigue was measured using the Chinese version (Åhsberg et al., 1997). The 25-item scale assesses lack of energy, physical exertion, physical discomfort, lack of motivation, and sleepiness on a 6-point scale. Higher scores reflect more severe fatigue. Cronbach’s *α* was 0.99 for the total scale. For the subscales, Cronbach’s *α* coefficients were as follows: lack of energy (*α* = 0.93), physical exertion (*α* = 0.92), physical discomfort (*α* = 0.91), lack of motivation (*α* = 0.92), and sleepiness (*α* = 0.94). The mean inter-item correlation for the total scale was 0.58, supporting internal consistency without excessive item redundancy (Clark and Watson, 1995). The SOFI-C has been previously validated in Chinese occupational samples, demonstrating adequate psychometric properties and cross-cultural measurement equivalence (Zhu et al., 2018).

#### Recovery experience questionnaire (REQ)

The REQ (Sonnentag and Fritz, 2007) evaluates off-job recovery quality. The 16-item questionnaire comprises four subscales: psychological detachment, relaxation, mastery, and control, rated on a 5-point scale. Higher scores indicate better recovery. Cronbach’s *α* was 0.97 for the total scale; subscale *α* ranged from 0.90 to 0.94. The mean inter-item correlation for the total scale was 0.60, within the recommended range (Clark and Watson, 1995). The Chinese version of the REQ has been validated in Chinese employee samples, demonstrating good factor structure and reliability (Zhang et al., 2019).

### Procedure and data analysis

#### Procedure

Following administrative and ethical approval, trained researchers collected data during centralized rest periods. Participants provided written informed consent after the study’s purpose, confidentiality, and voluntary nature were explained. Anonymous paper questionnaires were administered with researchers available for clarification. Completion time was approximately 20–25 min.

To minimize the potential for common method bias, several procedural remedies were implemented during data collection (Podsakoff et al., 2003). First, participants were assured of the anonymity and confidentiality of their responses, and they were informed that there were no right or wrong answers, thereby reducing evaluation apprehension and social desirability bias. Second, the questionnaire was designed with different scale formats and response anchors across the measures (e.g., 5-point vs. 6-point scales; Likert-type vs. frequency-based anchors) to reduce method-related variance. Third, the order of the scales was counterbalanced, and some items were reverse-scored to minimize response pattern biases. Fourth, the questionnaire was prefaced with instructions emphasizing the importance of honest responses and the voluntary nature of participation.

#### Data analysis

After screening and removing invalid data, SPSS 31.0 was used for descriptive statistics, common method bias testing, and correlations. Structural equation modeling (SEM) was performed using AMOS 28.0 to test the serial mediation model. The significance of mediation effects was tested using the bootstrap method with 5,000 resamples (95% bias-corrected confidence intervals). The significance level was set at *α* = 0.05.

## Results

### Common method Bias test

Given the cross-sectional, self-report design, common method bias (CMB) poses a potential threat to the validity of the findings (Podsakoff et al., 2003). To assess the extent of CMB, Harman’s single-factor test and a common latent factor analysis were conducted based on the measurement model used in hypothesis testing (Anderson and Gerbing, 1988).

First, Harman’s single-factor test was performed by entering all items from the final measurement model into an unrotated exploratory factor analysis. The results revealed that multiple factors with eigenvalues greater than 1 emerged, and the first factor accounted for 32.4% of the total variance, which is below the recommended threshold of 40% (Podsakoff et al., 2003; Fuller et al., 2016). This suggests that no single factor explains the majority of the variance, indicating that common method bias is unlikely to be a serious concern.

Second, the common latent factor (CLF) approach was employed to further assess the presence of CMB (Podsakoff et al., 2003; Williams et al., 1989). A latent method factor was added to the measurement model, with all items loading on both their theoretical constructs and the method factor. The fit of this model was then compared with the original measurement model, and the significance of path coefficients was examined. The model including the CLF showed a slight improvement in fit (ΔCFI < 0.01), and the average variance explained by the method factor was 15%, well below the 20% guideline recommended by Williams et al. (1989). Importantly, the pattern and significance of all factor loadings and inter-construct correlations remained unchanged after controlling for the CLF, indicating that the method factor did not substantially influence the relationships among the substantive constructs.

Third, the standardized regression weights of the measurement model with and without the CLF were compared. The differences between the weights were all less than 0.20, with an average difference of 0.03, further supporting that common method bias does not threaten the validity of the conclusions (Cote and Buckley, 1987).

Collectively, these diagnostic tests suggest that common method bias is not a significant concern in this study, and the observed relationships among the constructs reflect substantive associations rather than methodological artifacts.

### Measurement model evaluation

Prior to testing the structural model, a series of confirmatory factor analyses (CFAs) were conducted to evaluate the psychometric properties of the multi-item scales in this specific occupational sample. All CFAs were performed using AMOS 28.0 with maximum likelihood estimation. Model fit was assessed using multiple indices: the comparative fit index (CFI), Tucker–Lewis index (TLI), root mean square error of approximation (RMSEA) with 90% confidence intervals, and standardized root mean square residual (SRMR). Acceptable model fit was defined as CFI/TLI ≥ 0.90, RMSEA ≤ 0.08, and SRMR ≤ 0.08 (Hu and Bentler, 1999).

For anxiety sensitivity (ASI-3), a three-factor model representing the three theorized dimensions (physical, cognitive, and social concerns) was tested. The model demonstrated acceptable fit to the data: *χ*^2^/*df* = 2.45, CFI = 0.94, TLI = 0.93, RMSEA = 0.06 (90% CI [0.05, 0.07]), SRMR = 0.05. All factor loadings were significant (*p* < 0.001) and exceeded 0.60. Average variance extracted (AVE) ranged from 0.52 to 0.60, and composite reliability (CR) ranged from 0.85 to 0.90, indicating adequate convergent validity (Fornell and Larcker, 1981).

For recovery experience (REQ), a four-factor model (psychological detachment, relaxation, mastery, and control) was tested. The model showed good fit: *χ*^2^/*df* = 2.21, CFI = 0.96, TLI = 0.95, RMSEA = 0.05 (90% CI [0.04, 0.06]), SRMR = 0.04. Standardized factor loadings ranged from 0.65 to 0.89 (all *p* < 0.001). AVE values ranged from 0.55 to 0.68, and CR values ranged from 0.88 to 0.96, supporting convergent validity.

For emotion regulation self-efficacy (RESE), a three-factor model (positive affect, despondency/distress, and anger/irritation) was tested. The model demonstrated excellent fit: *χ*^2^/*df* = 1.89, CFI = 0.98, TLI = 0.97, RMSEA = 0.04 (90% CI [0.03, 0.05]), SRMR = 0.03. All factor loadings exceeded 0.70 (*p* < 0.001). AVE ranged from 0.60 to 0.68, and CR ranged from 0.92 to 0.96, indicating strong convergent validity.

Discriminant validity was assessed using the Fornell–Larcker criterion (Fornell and Larcker, 1981), which requires that the square root of AVE for each construct exceed its correlations with other constructs. The square roots of AVE (ranging from 0.72 to 0.82) were greater than all corresponding inter-construct correlations, providing evidence of discriminant validity. Additionally, the hypothesized measurement models were compared with alternative one-factor models (where all items were loaded onto a single factor); the one-factor models exhibited significantly poorer fit (e.g., for ASI-3: *χ*^2^/*df* = 6.82, CFI = 0.62, RMSEA = 0.18), confirming that common method variance does not account for the observed factor structures (Podsakoff et al., 2003).

These results confirm that all scales possess adequate psychometric properties in this sample of meteorological exploration personnel, supporting their use in subsequent structural analyses.

### Descriptive statistics and correlation analysis

Means, standard deviations, and intercorrelations are presented in Table 1. Anxiety sensitivity (*M* = 2.57, *SD* = 0.99) and occupational fatigue (*M* = 2.55, *SD* = 1.02) scores were in the moderate range. Recovery experience (*M* = 3.24, *SD* = 1.00) and emotion regulation self-efficacy (*M* = 3.28, *SD* = 1.07) were slightly above the theoretical midpoint.

**Table 1:** Descriptive statistics and correlations among study variables (*n* = 311).

Variable	*M*	*SD*	1	2	3	4	5
1. Gender	1.07	0.25					
2. Age	2.17	0.50	−0.24*				
3. AS	2.57	0.99	0.00	−0.11			
4. REXP	3.24	1.00	0.08	0.03	0.46**		
5. RESE	3.28	1.07	0.05	0.11	0.38**	0.91**	
6. OF	2.55	1.02	0.10	−0.12	0.86**	0.45**	0.35**

*p < 0.05, **p < 0.01. Gender was coded as 1, Male; 2, Female. Age was coded into brackets (1–5). AS, Anxiety Sensitivity; REXP, Recovery Experience; ERSE, Emotion Regulation Self-Efficacy; OF, Occupational Fatigue.

A strong positive correlation was found between anxiety sensitivity and occupational fatigue (*r* = 0.86, *p* < 0.001), supporting Hypothesis 1. Anxiety sensitivity was positively correlated with recovery experience (*r* = 0.46, *p* < 0.001) and emotion regulation self-efficacy (*r* = 0.38, *p* < 0.001). Recovery experience and emotion regulation self-efficacy were highly correlated (*r* = 0.91, *p* < 0.001). Both recovery experience (*r* = 0.45, *p* < 0.001) and emotion regulation self-efficacy (*r* = 0.35, *p* < 0.001) were positively correlated with occupational fatigue. Correlations between demographic variables (gender, age) and core study variables were weak; thus, they were not included as controls in subsequent analyses. Notably, the positive associations between AS and RE, and between RE and OF, appear counterintuitive from a traditional resource perspective but may reflect the phenomenon of the “recovery paradox” (Sonnentag, 2018), wherein recovery efforts in chronically stressed populations can be ineffective or even resource-depleting.

### Sensitivity analysis for gender

Although the gender distribution accurately reflects the occupational context, the small number of female participants (*n* = 14) precluded meaningful gender-stratified analyses or tests of moderation by gender. To assess whether gender influenced the main model parameters, a sensitivity analysis was conducted by including gender as a control variable in the structural model. The path coefficients and indirect effects remained virtually unchanged after controlling for gender (e.g., the direct effect of AS on OF changed from *β* = 0.84 to *β* = 0.83, both *p* < 0.001), indicating that the model parameters are robust to gender effects (Becker et al., 2016).

### Tests of main, mediating, and chain mediating effects

**Figure 1:**
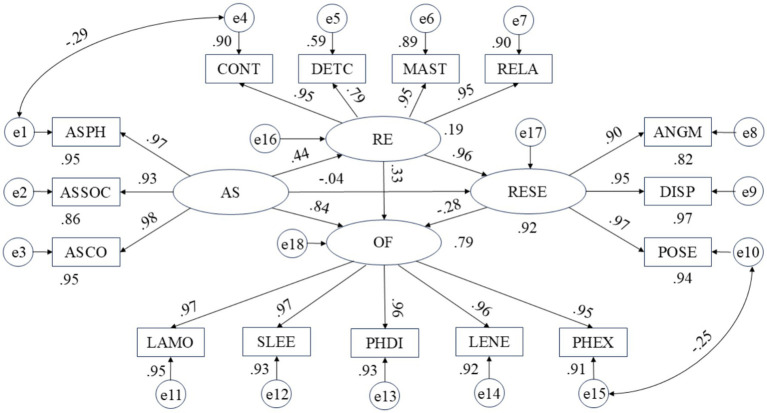
Final serial mediation model with standardized path coefficients (*n* = 311).

To examine the hypothesized mediating roles of recovery experience and emotion regulation self-efficacy, the serial mediation model was tested using structural equation modeling (SEM) in AMOS 28.0. The model specified anxiety sensitivity (AS) as the exogenous variable, recovery experience (RE) and emotion regulation self-efficacy (ERSE) as sequential mediators, and occupational fatigue (OF) as the outcome variable. Following the theoretical framework of Sonnentag and Fritz (2007), RE was operationalized as a four-dimensional construct comprising psychological detachment, relaxation, mastery, and control. All latent variables were measured by their respective subscale scores as observed indicators. The final model with standardized path estimates is presented in Figure 1.

The model demonstrated acceptable fit to the data: *χ*^2^/*df* = 2.36, CFI = 0.94, TLI = 0.93, RMSEA = 0.09 (90% CI [0.08, 0.10]), SRMR = 0.06. Although the RMSEA value slightly exceeded the conventional cutoff of 0.08 recommended by Hu and Bentler (1999), the remaining fit indices met the criteria for acceptable model fit. Given the theoretical importance of retaining the full recovery experience construct—particularly the psychological detachment dimension, which is central to the recovery process (Sonnentag and Fritz, 2007)—theoretical fidelity was prioritized over purely statistical considerations, and this theoretically specified model was retained for hypothesis testing (MacCallum et al., 1992). The complete fit indices are reported in Table 2.

**Table 2:** Fit indices for the serial mediation model (*n* = 311).

Fit index	Criterion	Model result	Evaluation
PCMIN/DF	1 ~ 3	2.36	Good
RMSEA	<0.08	0.09 (90% CI [0.08, 0.10])	Marginal
GFI	>0.90	0.97	Good
NFI	>0.90	0.95	Good
RFI	>0.90	0.94	Good
IFI	>0.90	0.98	Good
TLI	>0.90	0.93	Good
CFI	>0.90	0.94	Good

Although the RMSEA slightly exceeds the conventional cutoff of 0.08, the other fit indices meet the criteria for acceptable fit. Given the theoretical importance of retaining the full recovery experience construct, we prioritized theoretical fidelity over purely statistical considerations (MacCallum et al., 1992).

The standardized path coefficients, along with their standard errors and significance levels, are displayed in Figure 1 and summarized as follows: AS was positively and significantly associated with RE (*β* = 0.42, *SE* = 0.15, *p* < 0.05). RE, in turn, was a strong positive predictor of ERSE (*β* = 0.97, *SE* = 0.08, *p* < 0.001). ERSE was negatively associated with OF (*β* = −0.23, *SE* = 0.10, *p* < 0.05). The direct association between AS and OF remained significant and positive (*β* = 0.84, *SE* = 0.12, *p* < 0.001), indicating that the relationship between AS and OF is partially mediated by RE and ERSE, because the direct effect remains significant after inclusion of the mediators.

To test the significance of the indirect effects, a bias-corrected bootstrap procedure with 5,000 resamples was employed. The results revealed that the indirect effect of AS on OF through RE alone was significant (*β* = 0.126, *SE* = 0.042, 95% CI [0.021, 0.352]), supporting Hypothesis 2. The indirect effect through ERSE alone was also significant (*β* = 0.007, *SE* = 0.003, 95% CI [0.045, 0.398]), supporting Hypothesis 3. Crucially, the serial indirect effect through RE and then ERSE was significant and negative (*β* = −0.094, *SE* = 0.030, 95% CI [−0.361, −0.058]), confirming Hypothesis 4. This negative serial mediation arises because ERSE negatively predicts OF, while AS positively predicts RE and RE positively predicts ERSE; thus, the product of these paths is negative, indicating that higher AS is associated with lower OF through the RE → ERSE pathway, though this effect is small in magnitude and should be interpreted in the context of the large positive direct effect.

The total effect of AS on OF was 0.879. After including RE and ERSE as mediators, the direct effect was 0.842, accounting for 95.6% of the total effect, while the total indirect effect accounted for the remaining 4.4%. Within the total indirect effect, the specific path via RE contributed 14.3%, the serial mediation path contributed −10.7%, and the path via ERSE contributed 0.8%. These findings indicate that although the relationship between anxiety sensitivity and occupational fatigue is predominantly direct, significant indirect pathways exist through recovery experience and emotion regulation self-efficacy, both independently and sequentially.

## Discussion

### Main effect of anxiety sensitivity on fatigue

Hypothesis 1 was confirmed: anxiety sensitivity significantly and positively predicted occupational fatigue. This finding aligns with prior research linking AS to exhaustion and performance impairment in high-stress occupations (Taylor et al., 2007; Bardeen and Fergus, 2016). The relationship can be understood through a multi-level resource perspective. Cognitively, catastrophic misinterpretation of arousal signals leads to perseverative worry and rumination, consuming attentional resources and contributing to mental exhaustion (Bardeen and Fergus, 2016). Physiologically, anxiety sensitivity triggers frequent stress responses, disrupting cortisol rhythms and sleep architecture (Chou et al., 2018), thereby potentially undermining daily recovery. Maladaptive emotion regulation strategies often employed by high-AS individuals further accelerate self-regulatory resource depletion (Tamir et al., 2020). Anxiety-driven concerns can also prevent genuine psychological detachment during off-job time, turning recovery into a resource-depleting experience (Sonnentag, 2018). Collectively, anxiety sensitivity is a key factor associated with the dynamic imbalance of resource loss and recovery (Hobfoll, 1989), underscoring the need for interventions that target anxiety sensitivity through integrated approaches such as cognitive restructuring, sleep management, and emotion regulation training.

Contrary to some prior studies, recovery experience in this sample showed positive correlations with both anxiety sensitivity and fatigue. This may reflect the unique context of meteorological exploration, characterized by chronically high-risk and uncertain environments that foster sustained hypervigilance. For these individuals, recovery efforts may frequently be undermined by an inability to achieve psychological detachment, rendering them ineffective and frustrating, thus transforming recovery from a resource-replenishing activity into a process associated with further depletion. This pattern aligns with the concept of the recovery paradox (Sonnentag, 2018), which posits that under conditions of high stress, recovery attempts may paradoxically become sources of additional strain, resulting in positive correlations between recovery experiences and fatigue. This finding highlights the situational specificity and complexity of recovery within this occupational group.

### Mediating role of recovery experience

Hypothesis 2 was supported: recovery experience mediated the relationship between anxiety sensitivity and occupational fatigue, indicating that it may not always function protectively but can serve as a conduit through which anxiety sensitivity exacerbates fatigue. This occurs primarily through impaired psychological detachment. The hypervigilance and rumination characteristic of high anxiety sensitivity prevent individuals from disengaging mentally from work-related concerns during leisure time—a state termed psychological detachment failure (Bardeen and Fergus, 2016). Traits that consume cognitive resources can erode recovery’s core replenishment function (Sonnentag and Bayer, 2005). Failed recovery attempts may initiate a vicious cycle of recovery frustration, where the inability to relax induces feelings of failure and helplessness, paradoxically creating a new source of stress and resource loss (Sonnentag, 2018). These results resonate with research highlighting the interaction between personal traits and occupational context in determining recovery outcomes (Sonnentag et al., 2017). They clarify that anxiety sensitivity is a key individual trait that can invert recovery’s typical protective role. Consequently, for high-AS personnel, interventions should extend beyond providing rest time to focus on enhancing recovery quality. Strategies such as cognitive-behavioral training to improve detachment skills or mindfulness-based relaxation could help restore recovery’s resource-replenishing function.

### Mediating role of emotion regulation self-efficacy

Support was found for Hypothesis 3: emotion regulation self-efficacy mediated the relationship between anxiety sensitivity and fatigue, underscoring confidence in one’s ability to manage emotions as a critical psychological mechanism. Individuals high in anxiety sensitivity often possess lower perceived self-efficacy for emotion regulation due to their fear of anxiety sensations (Taylor et al., 2007). This deficit is associated with a tendency to rely on maladaptive strategies such as avoidance or perseverative worry when facing fieldwork pressures, leading to inefficient, resource-consuming regulation attempts (Tamir et al., 2020). Conversely, strong emotion regulation self-efficacy acts as a positive belief resource, increasing regulation efficiency and reducing perceived effort, thereby conserving psychological resources (Bandura, 1997). For personnel whose baseline resources are already depleted by chronic vigilance, low self-efficacy may exacerbate the internal resource drain when coping with anxiety. This finding aligns with research identifying emotion regulation self-efficacy as a key predictor of mental health outcomes under stress (Caprara et al., 2008) and confirms that boosting regulatory confidence is a viable intervention target. Therefore, interventions should incorporate training designed to enhance emotion regulation self-efficacy, utilizing cognitive-behavioral techniques to challenge inefficacy beliefs and build mastery through guided practice of adaptive strategies such as cognitive reappraisal.

### Serial mediation pathway

Hypothesis 4 was validated: a significant negative serial mediation effect was found through recovery experience and emotion regulation self-efficacy, illuminating a consumptive psychological sequence. High anxiety sensitivity is associated with low-quality recovery, which is associated with reduced regulatory confidence, ultimately exacerbating fatigue. The pathway begins with anxiety sensitivity eroding recovery quality by impeding psychological detachment (Sonnentag and Bayer, 2005) and deep relaxation (Chou et al., 2018), resulting in ineffective recovery that incurs net resource loss (Sonnentag, 2018). Subsequently, this low-quality recovery fails to provide mastery experiences. While successful recovery can bolster confidence in self-regulation (Molino et al., 2015), repeated ineffective recovery delivers persistent negative feedback, systematically eroding belief in one’s emotion management capabilities and lowering self-efficacy. Finally, diminished self-efficacy is related to increased fatigue by making individuals more prone to maladaptive, resource-intensive coping strategies such as catastrophizing (Tamir et al., 2020). This integrated model resonates with concepts such as the recovery paradox (Sonnentag, 2018) and the person-situation contingency of recovery benefits (Sonnentag et al., 2017). This study is the first to empirically demonstrate this complete “trait–process–belief–outcome” negative transmission chain in meteorological exploration personnel, revealing how protective psychological factors can transform into risk factors under sustained high pressure. The key implication is that for high-AS personnel, simply increasing rest time or conducting isolated emotion regulation training may be insufficient. A systematic, sequential intervention approach is suggested: first, using cognitive training to reduce anxiety sensitivity and rumination to improve recovery quality; second, leveraging successful recovery experiences to consolidate emotion regulation confidence; and finally, utilizing this enhanced efficacy to achieve resource conservation, thereby breaking the vicious cycle of fatigue.

The negative sign of the serial indirect effect is mathematically a consequence of the negative path from ERSE to OF combined with the positive paths from AS to RE and RE to ERSE. Conceptually, this suggests that while high AS generally increases fatigue directly, it also triggers a resource-depletion cascade (impaired recovery → lower regulatory self-efficacy) that paradoxically results in a slight counterbalancing effect. However, given its small magnitude relative to the direct effect, the dominant mechanism remains the direct detrimental influence of anxiety sensitivity on occupational fatigue. The significant serial mediation nonetheless highlights the theoretical importance of the recovery–self-efficacy pathway as a secondary process.

### Theoretical contributions and integration of COR and self-efficacy theories

The findings of this study offer important theoretical contributions by empirically validating the integrated framework proposed in the Introduction, which combines Conservation of Resources (COR) theory and Bandura’s Self-Efficacy Theory. This integration not only clarifies the division of explanatory labor between the two theories but also demonstrates their complementarity in explaining the complex process linking anxiety sensitivity to occupational fatigue.

First, consistent with COR theory’s emphasis on resource depletion and loss spirals (Hobfoll, 1989, 2001), the results show that high anxiety sensitivity (AS) is negatively associated with recovery experience (RE) by consuming cognitive and emotional resources necessary for effective psychological detachment and relaxation (the AS→RE link). Furthermore, the finding that poor recovery is negatively associated with emotion regulation self-efficacy (the RE → ERSE link) aligns with the COR theory concept of loss spirals: failed resource investment (ineffective recovery) leads to further resource depletion, specifically eroding a key personal resource—regulatory confidence. This pattern corroborates recent work by Hoover et al. (2022), who demonstrated that resource-depleted individuals struggle to benefit from recovery opportunities, and extends this logic to show how such depletion cascades into diminished self-beliefs.

Second, in line with Bandura’s Self-Efficacy Theory (Bandura, 1997), the results confirm that ERSE serves as a critical proximal determinant of occupational fatigue (the ERSE→OF link). Individuals with low confidence in their emotion regulation capabilities are more likely to engage in maladaptive, resource-intensive coping strategies, accelerating resource depletion and fatigue accumulation (Shoji et al., 2016; Xanthopoulou et al., 2013). This finding reinforces the agentic perspective of social cognitive theory, emphasizing that beliefs about one’s capabilities are not merely passive reflections of past experiences but actively shape future outcomes.

The integration of these two theories yields explanatory power that exceeds what either framework could provide alone. COR theory alone, while adept at explaining resource dynamics, does not fully account for the specific cognitive and behavioral mechanisms through which depleted resources translate into sustained fatigue. Self-Efficacy Theory alone, while illuminating the role of agentic beliefs, does not explain why these beliefs become depleted in the first place. By combining the two frameworks, the model captures the full trajectory from a stable vulnerability trait (AS), through a resource-depleting process (impaired RE), to a compromised belief system (low ERSE), and finally to a negative health outcome (OF). This integrated approach responds to recent calls in the occupational health psychology literature for multi-theoretical models that bridge resource-based and cognitive-behavioral perspectives (Halbesleben et al., 2014; Luszczynska and Schwarzer, 2015).

Moreover, the predominance of the direct effect of AS on OF (95.6% of the total effect) does not diminish the theoretical importance of the indirect pathways. Rather, it suggests that anxiety sensitivity may be related to fatigue through multiple routes: a rapid, direct route that may operate through immediate cognitive intrusions and physiological hyperarousal, and a slower, erosive route that unfolds through compromised recovery and eroded self-efficacy. This multi-route conceptualization is consistent with contemporary perspectives on trait influences on well-being, which recognize that stable dispositions can affect outcomes through both direct and mediated pathways (Bolger and Zuckerman, 1995; Taylor et al., 2020).

### Interpretation of the high direct effect and its implications

The finding that the direct effect of anxiety sensitivity on occupational fatigue accounted for the majority of the total effect (95.6%) warrants careful interpretation. This robust direct pathway highlights anxiety sensitivity as a potent and proximate vulnerability factor in this population. It suggests that, within the chronically demanding context of meteorological exploration, the association of this trait with fatigue may be partly mediated through rapid, resource-intensive processes not fully captured by the model. These could include immediate cognitive intrusions, sustained hypervigilance, or heightened somatic awareness that directly drain energy and motivation, aligning with the concept of “trait-like” direct effects on strain outcomes (Hobfoll, 1989).

*Theoretically*, the significant serial mediation, despite its smaller proportion, remains crucial. It validates a specific, theory-driven mechanism whereby anxiety sensitivity impairs recovery quality, which in turn undermines regulatory self-efficacy, thereby contributing to fatigue. This pattern supports a multi-route model of trait influence, where a dominant direct effect coexists with specific indirect psychological pathways. It enriches understanding by moving beyond a simple correlation to delineate *how* the trait might also exert a slower, erosive influence through key recuperative and belief processes.

*Practically*, this pattern informs intervention priorities. The strong direct effect underscores that primary prevention should target anxiety sensitivity itself, potentially through cognitive-behavioral approaches that reduce catastrophic appraisals of arousal. Concurrently, the validated indirect pathways identify precision intervention targets for secondary prevention. For individuals with high anxiety sensitivity, enhancing true psychological detachment and bolstering emotion regulation self-efficacy represent viable strategies to mitigate the additional fatigue risk conveyed through these specific channels. Thus, a tiered intervention approach—addressing the core vulnerability while also strengthening recovery and regulatory resources—is suggested by these findings.

### Practical implications

The findings of this study offer actionable insights for developing targeted interventions to mitigate occupational fatigue among meteorological exploration personnel. Rather than proposing generic programs, specific intervention strategies were mapped onto each pathway identified in the serial mediation model, thereby translating empirical evidence into a coherent, pathway-based intervention framework.

*For the AS → RE pathway* (anxiety sensitivity → impaired recovery experience), interventions should aim to reduce the cognitive and emotional resource drain that hinders effective psychological detachment. Mindfulness-based interventions, such as Mindfulness-Based Stress Reduction (MBSR; Kabat-Zinn, 1990), have been shown to diminish hypervigilance and rumination—core features of high anxiety sensitivity (Gu et al., 2015). Adapted for field personnel, brief mindfulness exercises or detachment training delivered during rest periods could help individuals disengage from work-related concerns, thereby improving recovery quality. Such practices may interrupt the persistent attentional focus on anxiety-related cues and facilitate genuine psychological detachment (Hülsheger et al., 2014).

*For the RE → ERSE pathway* (poor recovery → eroded regulatory self-efficacy), interventions should focus on providing mastery experiences within recovery activities. According to Social Cognitive Theory (Bandura, 1997), self-efficacy is strengthened by successful performance. Designing recovery activities that offer achievable challenges and opportunities for skill mastery—such as engaging in competence-affirming hobbies, structured physical activities with progressive goals, or problem-solving tasks during off-duty time—could rebuild confidence in one’s capacity to manage emotions. For instance, adventure-based learning or team-based recreational programs that foster a sense of accomplishment may enhance emotion regulation self-efficacy (Molino et al., 2015).

*For the ERSE → OF pathway* (low regulatory self-efficacy → increased fatigue), cognitive-behavioral techniques aimed at enhancing emotion regulation skills are recommended. Cognitive restructuring, a core component of Cognitive-Behavioral Therapy (CBT; Beck, 2011), can help individuals challenge catastrophic interpretations of emotional experiences and develop more adaptive coping strategies. Training programs that teach cognitive reappraisal and problem-focused coping have been shown to reduce emotional exhaustion and fatigue (Richardson and Rothstein, 2008). Such interventions could be delivered in group workshops or through digital platforms, tailored to the high-demand context of meteorological exploration.

Importantly, these interventions should not be viewed in isolation. A comprehensive, multi-component program that sequentially targets each pathway—first reducing anxiety-driven resource depletion (AS→RE), then rebuilding self-efficacy through mastery experiences (RE → ERSE), and finally strengthening emotion regulation skills (ERSE→OF)—may yield synergistic benefits. Future research should test the efficacy of such integrated approaches, ideally using randomized controlled designs, to establish causal evidence for these pathway-specific recommendations. By grounding intervention design in the empirical mechanisms revealed by the model, practitioners can move toward precision-based mental health promotion in high-risk occupational settings.

### Limitations and future directions

Several limitations of this study provide directions for future research.

First, the cross-sectional design of this study precludes any definitive causal inferences. Although the theoretical model posits directional relationships among anxiety sensitivity, recovery experience, emotion regulation self-efficacy, and occupational fatigue, the data only establish associations at a single time point. It is well established in the methodological literature that cross-sectional data, even when analyzed with structural equation modeling, cannot rule out reverse causality or establish temporal precedence (Segerstrom and O’Connor, 2012). For instance, it is possible that prolonged occupational fatigue erodes recovery capacity and self-efficacy beliefs, rather than the reverse. Future research should employ longitudinal or experience-sampling designs to capture the dynamic, within-person processes over time and to better understand the temporal ordering of these variables.

Second, while the hypothesized serial mediation was statistically supported, the high proportion of the direct effect (95.6%) indicates that other significant mediators likely exist. Future research should explore additional mechanistic pathways, such as sleep disturbance (which may directly translate physiological hyperarousal into fatigue), maladaptive coping behaviors (e.g., substance use), or work-specific cognitive processes like persistent safety-related rumination. Integrating these variables could yield a more comprehensive model explaining a greater share of the variance.

Third, the modest relative magnitude of the indirect effects invites investigation into potential moderators. Factors such as perceived organizational support, team cohesion, or job autonomy might buffer the impact of anxiety sensitivity on recovery or strengthen the protective role of self-efficacy. Testing such moderated-mediation models would clarify boundary conditions and help identify subgroups for whom interventions targeting recovery and self-efficacy would be most potent.

Fourth, the reliance on self-report measures, despite procedural and statistical controls for common method bias, may inflate variable relationships. Although Harman’s single-factor test (first factor = 32.4%) and common latent factor analysis (method factor explained 15% of variance) suggested that common method bias is not a serious concern, future research should incorporate objective measures to enhance robustness. These could include actigraphy for sleep and recovery, physiological indices of stress reactivity (e.g., cortisol levels, heart rate variability), or supervisor-rated performance indicators.

Fifth, although the Chinese versions of the scales used in this study have been previously validated and demonstrated adequate psychometric properties in the sample (as reported in the Measurement Model Evaluation section), the cross-cultural measurement equivalence of these instruments across different occupational and cultural contexts warrants further investigation. While the SOFI-C has been validated in Chinese samples (Zhu et al., 2018), and the ASI-3 (Wang et al., 2011), RESE (Yu et al., 2009), and REQ (Zhang et al., 2019) have been adapted for Chinese populations, future research should formally test measurement invariance across countries and occupational groups to ensure that construct meanings are comparable (Vandenberg and Lance, 2000). This would strengthen the cross-cultural generalizability of the findings.

Sixth, the gender imbalance in the sample (95.5% male) warrants careful consideration. While this distribution accurately reflects the demographic composition of meteorological field exploration in China, where the physically demanding and remote nature of the work results in a predominantly male workforce (Li et al., 2020; Wang and Zhang, 2018), it limits the generalizability of the findings to female personnel. Given established gender differences in anxiety sensitivity (Stewart and Watt, 2008; Norr et al., 2015), recovery experiences, and fatigue susceptibility, future research should include more balanced samples to examine potential gender differences and test whether the proposed model holds for female exploration personnel. Sensitivity analysis including gender as a control variable indicated that the model parameters are robust to gender effects (Becker et al., 2016), but this does not substitute for adequately powered gender-stratified analyses.

Seventh, the geographic homogeneity of the sample represents another limitation. All participants were recruited from field exploration teams affiliated with a single provincial Meteorological Bureau and Geological Survey Bureau. This geographically concentrated sampling strategy introduces contextual homogeneity in terms of local climate conditions, institutional management culture, organizational work norms, and regional socioeconomic factors—all of which may moderate the relationships among the study variables (Taris and Schaufeli, 2015). Consequently, the generalizability of the findings to meteorological exploration personnel operating in other provinces of China, or in international field contexts with different environmental and organizational characteristics, remains uncertain. Future research should adopt multi-provincial or cross-national sampling strategies to test the robustness and cross-contextual applicability of the proposed model (Spector et al., 2019).

## Conclusion

This study yields several key conclusions. First, anxiety sensitivity significantly and positively predicted occupational fatigue, indicating that in high-pressure field environments, an individual’s tendency toward catastrophic interpretation of anxiety-related cues serves as a critical psychological risk factor exacerbating exhaustion. The dominant direct path underscores that interventions should prioritize mitigating anxiety sensitivity at the cognitive source. Second, recovery experience significantly mediated this relationship. Among highly anxiety-sensitive individuals, recovery is often marked by psychological detachment failure and ineffective effort, transforming it into a resource-depleting experience. This highlights that recovery quality is more consequential than its duration. Third, emotion regulation self-efficacy also played a partial mediating role. Insufficient confidence in one’s ability to manage emotions depletes psychological resources in this population, accelerating fatigue accumulation, pointing to the buffering value of regulatory confidence. Fourth, a significant serial mediating pathway was identified linking recovery experience and emotion regulation self-efficacy, with a negative effect size. This reveals a deleterious transmission mechanism: high anxiety sensitivity is associated with poor recovery quality, which is associated with lower confidence in emotion regulation; diminished confidence is in turn associated with increased fatigue.

By focusing on an extreme high-stress professional cohort, this research is the first to demonstrate that recovery experience can shift from a protective factor to a consumptive mediator—a context-dependent mechanism. The findings not only confirm the explanatory power of COR Theory and Self-Efficacy Theory in exceptional environments but, more importantly, elucidate through a serial mediation model the dynamic transformation path from trait to process, to belief, and finally to outcome. This underscores the complexity of psychological mechanisms under the interaction between individual traits and occupational context, offering a novel theoretical lens for understanding multi-stage fatigue formation in high-pressure populations. Consequently, interventions must be systematically designed: first, reduce anxiety sensitivity through cognitive training; second, enhance recovery effectiveness via mindfulness and related methods; and third, build emotion regulation self-efficacy through skill training and accumulated success experiences. Such an integrated strategy—spanning cognition, behavior, and belief—can help disrupt the vicious cycle of fatigue and provides an empirical foundation for safeguarding mental health and operational safety in this professional group.

## Data Availability

The datasets presented in this study can be found in online repositories. The names of the repository/repositories and accession number(s) can be found in the article/supplemental material.

## Glossary

ANGM: Management of Anger/Irritation
AS: Anxiety Sensitivity
ASCO: Cognitive Concerns
ASPH: Physical Concerns
ASSOC: Social Concerns
CONT: Control
COR: Conservation of Resources Theory
DETC: Psychological Detachment
DISP: Management of Despondency/Distress
ERSE: Emotion Regulation Self-Efficacy
LAMO: Lack of Motivation
LENE: Lack of Energy
MAST: Mastery
OF: Occupational Fatigue
PHDI: Physical Discomfort
PHEX: Physical Exertion
POSE: Expression of Positive Affect
RELA: Relaxation
REXP: Recovery Experience
SEM: Structural Equation Modeling
SET: Self-Efficacy Theory
SLEE: Sleepiness
